# Bert-Enhanced Text Graph Neural Network for Classification

**DOI:** 10.3390/e23111536

**Published:** 2021-11-18

**Authors:** Yiping Yang, Xiaohui Cui

**Affiliations:** Key Laboratory of Aerospace Information Security and Trusted Computing, Ministry of Education, Wuhan University, Wuhan 430000, China; yangyiping@whu.edu.cn

**Keywords:** text classification, Bert, graph neural networks

## Abstract

Text classification is a fundamental research direction, aims to assign tags to text units. Recently, graph neural networks (GNN) have exhibited some excellent properties in textual information processing. Furthermore, the pre-trained language model also realized promising effects in many tasks. However, many text processing methods cannot model a single text unit’s structure or ignore the semantic features. To solve these problems and comprehensively utilize the text’s structure information and semantic information, we propose a Bert-Enhanced text Graph Neural Network model (BEGNN). For each text, we construct a text graph separately according to the co-occurrence relationship of words and use GNN to extract text features. Moreover, we employ Bert to extract semantic features. The former part can take into account the structural information, and the latter can focus on modeling the semantic information. Finally, we interact and aggregate these two features of different granularity to get a more effective representation. Experiments on standard datasets demonstrate the effectiveness of BEGNN.

## 1. Introduction

Text classification is a fundamental task in natural language processing. It aims to assign labels to natural language text. Text classification has wide range of application scenarios, such as in sentiment classification and question answering [1,2]. Among these related tasks, text classification is the core of their application. It is used to deal with complex text information, which provides great help for fast and accurate text mining. For example, in a sentiment classification task, we focus on sentiment related words and classify texts by establishing a special emotion related dictionary. In the selective question answering, we extract the features of questions and alternative answers and classify them to select the most appropriate answer. Since the text is unstructured data written in natural language, which brings certain difficulties to its classification.

Early classification methods used bag-of-words features [3], that is, to calculate which word appeared in the text, and take it as the representation of the text, but this method did not consider the context information. The last decade has witnessed significant advances in text feature extraction using deep neural networks. Recently, with the progress in artificial intelligence research, a large number of neural network-based models are widely used in the task of text classification. Pre-trained word vectors such as Word2Vec [4] provide better initial embeddings for the tokens in the sentences. Other models such as RNN [5] and TextCNN [6] have also been proven effective in processing text data. In recent years, the pre-trained language model Bert [7] has gained increased attention and has refreshed the records in multiple natural language processing tasks. Attention mechanism [8] has also been integrated into various deep learning methods, which greatly improves the classification accuracy.

However, these methods cannot model the local and global structure features in text. While GNN has natural advantages in modeling structural information. There have been studies using graph neural networks to model text data. Some works build homogeneous graphs or heterogeneous graphs from text data and perform graph neural network propagation such as convolution operations on the graphs [9,10]. In this way, the model can take into account the structural information, which is of great significance for understanding the meaning of the text. However, some methods build text graphs on the entire dataset, weakening the individual features of each document.

Based on the above analysis, the existing text classification methods have some limitations in text feature extraction. First, most models use RNN, LSTM [5] and other methods to process serialized data, which cannot take into account the text structure information. Secondly, some methods based on graph neural networks extract the representation of text by building a heterogeneous graph structure for the entire dataset, but it’s hard to consider a single text’s semantic features. In addition, some methods have combined structural features and semantic features of sequences for extraction, but they can not consider single text features alone or do not consider the interaction between features, which limits their representation ability.

To solve the problems of these algorithms, we construct the BEGNN model. Specifically, we first construct a graph structure for each document separately. Moreover, we propose to aggregate the features extracted from Bert and the features extracted by graph structures. The former represents the semantic information of the documents, and the latter is a representation that considers the structural feature of the text. Compared with other work, we also add a co-attention module to solve the problem of interaction between features, and performed a variety of experiments to integrate the features, which can maximize the representation ability of the extracted features.

Our contribution is as follows:

(1) Our model can extract features of different granularities, from a pre-trained language model and graph neural networks for text representation. It not only takes into account the semantic information, and also the structural information, which improves the effect of the learned text representation.

(2) In order to prevent the two features from being separated during the prediction process, we have designed and performed experiments on co-attention modules as well as different aggregation methods, which can consider the interaction of the two representations and make full use of them to achieve better classification capabilities.

(3) The experiment results and analysis on four datasets demonstrate the effectiveness of BEGNN.

In the following paragraphs: Section 2 introduces researches about text classification methods related to our work, Section 3 illustrates the overall model we proposed, Section 4 shows the experimental results, and finally, the conclusion.

## 2. Related Work

### 2.1. Traditional Feature Engineering Method

Traditional text classification methods need to extract manually defined features, and they are often combined with machine learning algorithms for training and prediction. For a specific task, some early studies classify sentences or documents by analyzing text data and extracting statistical features of the text, then use pre-specified training set as training data. Bag-Of-Words (BOW) [11] and n-grams [12] are commonly used word-based representation method, which represents a sentence based on a collection of words or n-gram sequences which occur in it. These features are usually combined with models such as SVM [11] and have achieved good results. However, machine learning requires extensive feature engineering and relies on domain knowledge, which makes it difficult for the features on a single task to be generalized to other aspects.

### 2.2. Deep Learning-Based Method

Methods based on deep learning have been investigated to resolve the limitations of manual feature engineering [13,14]. The text is automatically mapped to a low-dimensional vector through the model to extract text features. Word embedding has brought new solutions to natural language tasks. Word2Vec [4] and GloVe [15] have been drawing great attention in NLP tasks. Mikolov et al. have shown these pre-trained embeddings can capture meaningful semantic features [16]. In addition, RNN models [17] have shown advantages in processing sequence data. TextCNN [6] performs convolution operations on text features and has achieved good results. Tan et al. [18] use a structure based on a dynamic convolutional gated neural network, making it possible to selectively control how much context information is contained in each specific location.

Recently, pre-trained language models have caused a great upsurge in research. Models such as Bert [7] are pre-trained on a large corpus, and can be simply transferred to downstream NLP tasks with fine-tuning, which have refreshed records on multiple NLP tasks. Bert [7] takes advantage of the self-attention mechanism, and builds a multi-layer self-attention network, which can also realize parallel computing. The attention mechanism is applied to various models, greatly improving the performance of various NLP tasks [19]. There have also been some studies exploring how to efficiently use Bert for natural language processing tasks [20,21,22]. These models have been proved effective in extracting features, but they cannot fully utilize the text’s structural features. While graph structure has natural advantages in modeling structural information.

There have been some researches that model text as graph structure for feature extraction. GNN [23] can capture the features of the nodes and the structural features in the graph, which can learn more effective representations for the nodes or the whole graph. GatedGNN [10] and GCN [9] have been applied to the task of text classification. Textgcn [9] constructed a heterogeneous graph network of words and documents, and uses co-occurrence features and TFIDF to measure the relationship between words and documents. For a new document, it needs to update the whole graph structure to perform prediction. Additionally, it cannot take into account the structural characteristics of a single document well. TextING [10] builds graph structure on each single text, which can learn the fine-grained word representation of the local structure. Lei et al. [24] designed a structure that can integrate the graph convolutional features of multi-layer neighbors, alleviating the problem of over-fitting to a certain extent. However, semantic features used in the models rely on pre-trained word embeddings, which limits the effect of the model. Parcheta et al. [25] studied the influence of embeddings extracted by combining different methods on text classification models.

There are also some methods that combine the pre-trained language model with graph neural networks to extract features. VGCN-Bert [26] builds a graph of the whole dataset, and uses the features extracted by GCN [27] to enhance the effect of Bert [7]. However, as in GCN, the unique structural characteristics of each text cannot be fully taken into account. Jeong et al. [28] simply concatenate the features of Bert and GCN for the recommendation task, but this method cannot consider the features’ interactive relationship, which reduces the representation ability. We show the methods of some related works in Table 1.

Considering the above problems, we propose to combine the features extracted by Bert [7] and graph neural networks, which can take into account the semantic and structural information of a single text. Different from the previous work, first of all, we build a graph structure for each text separately, and combine the graph neural network and Bert to extract different granular features. While most of the studies built a graph on the entire dataset or did not combine the different characteristics of different granularity. In addition, we employ the co-attention module to integrate features. As far as we know, we are the first to employ a co-attention module to combine the features of graph networks and Bert for text classification. So that we can take the advantages of the feature representation with different granularity.

## 3. Method

In this part, we describe the structure of BEGNN in detail.

### 3.1. Architecture Overview

**Figure 1:**
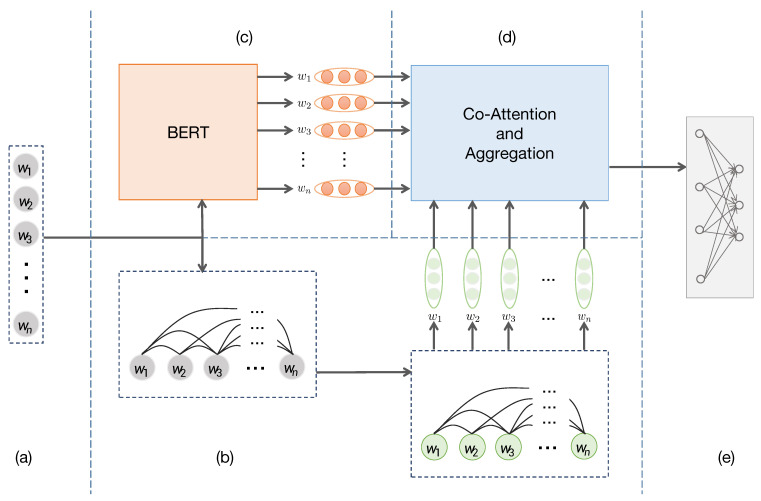
The architecture of BEGNN. (**a**) The input document. (**b**) Graph construction and graph neural network based feature extraction. (**c**) Bert based feature extraction. (**d**) Interactive feature aggregation. (**e**) Fully-connected layer.

The model structure is illustrated in Figure 1. BEGNN is composed of five modules: graph construction, Bert-based feature extraction, GNN based feature extraction, feature interaction and aggregation. Given a document represented as $W_{i}=\left\{w_{1},w_{2},\ldots ,w_{n}\right\}$, according to co-occurrence relationship of the words, we construct each text as a graph. By initializing the representation of graph nodes using word vectors and employing a graph neural network, we get the structure feature of each word. Moreover, We input the text into Bert [7] for semantic feature extraction. Finally, the two feature representations interact and aggregate through the co-attention layer and the aggregation layer to obtain the aggregated representation $H_{i}=\left\{h_{1},h_{2},\ldots ,h_{n}\right\}$. Taking the final representation $H_{i}$, we finally use the fully connected layer to predict the category. The details are presented in Section 3.2, Section 3.3, Section 3.4, Section 3.5 and Section 3.6, respectively.

### 3.2. Graph Construction

**Figure 2:**
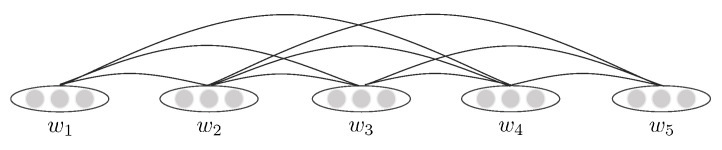
The graph constructed for a document with five words.

We create separate graphs for the documents, expressed as $G=\left(V,E\right)$. *V* is the set of nodes in the graph, including all the words in the text. While *E* includes edges between nodes. We use standard methods to pre-process the text, including word segmentation and cleaning. Afterwards, co-occurrence information is extracted to model the relationship between words in a document. We build an undirected text graph by setting a fixed-size sliding window, connecting the words appearing in the same window with undirected edges. Figure 2 is an instance.

The feature vector of the nodes are initialized with the GloVe word vector [15], document *i* is represented by $H_{i}^{0}=\left\{h_{1}^{0},h_{2}^{0},\ldots ,h_{\left|V\right|}^{0}\right\}$. $H_{i}^{0}\in R^{\left|V\right|*d}$, *d* is the word embedding size. For the graph structure established for each document, we use graph neural network for message passing.

### 3.3. Graph Neural Network Based Feature Extraction

For each text graph, we use Gated Graph Neural Networks [29] for feature propagation and extraction. It is a classical spatial domain message passing model based on GRU. The proposal of Gated Graph Neural Networks enables GNN to be better used to deal with sequence problems. In the process of message passing in the whole graph structure, the principle of GRU is adopted. The embedding of a node at time t+1 is determined by the embedding of itself and its neighbor nodes, and the edge information of the interaction between the nodes. By stacking such layers for *T* times, the nodes are able to receive the information of their *T*-hop neighbors. The formulas of the propagation recurrence in the *k*-th layer are:

(1) $$a^{t+1}=AH^{t}W_{a}+b$$

(2) $$z^{t+1}=\sigma \left(W_{z}a^{t+1}+U_{z}H^{t}+b_{z}\right)$$

(3) $$r^{t+1}=\sigma \left(W_{r}a^{t+1}+U_{r}H^{t}+b_{r}\right)$$

(4) $${\tilde{H}}^{t+1}=\tanh\left(W_{H}a^{t+1}+U_{H}\left(r^{t+1}⊙H^{t}\right)+b_{H}\right)$$

(5) $$H^{t+1}={\tilde{H}}^{t+1}⊙z^{t+1}+H^{t}⊙\left(1-z^{t+1}\right)$$

where $A\in R^{\left|V\right|*\left|V\right|}$ is the adjacency matrix, $a^{t+1}$ represents the result of the interaction between the nodes and their adjacent nodes through the edges. Formulas (2)–(4) is similar to the calculation process of GRU. Among them, $z^{t+1}$ controls the forgotten information, and $r^{t+1}$ controls the newly generated information. $H^{t+1}$ is the final updated node status of t+1-th layer. σ is sigmoid function. W, U and b are trainable weight matrices.

To simplify, we can write such a message passing process as:

(6) $$H^{t+1}=GGNN\left(H^{t},A;\Theta_{t}\right)$$

where $\Theta_{t}$ is the parameter set of the gated graph neural network of the *t*-th layer. After message passing of *T* layers, we get the final representation $H_{0}^{T}$.

### 3.4. Bert Based Feature Extraction

In addition to using GNN to obtain the features of the word nodes, we also fine-tune Bert to obtain the words’ semantic features. Pre-trained on large-scale corpus in an unsupervised way, the parameters of Bert are then fine-tuned according to downstream tasks. Bert is composed of the encoder of transformer module, which includes the self-attention layer and feed-forward layer. Self-attention is calculated by:

(7) $$Attention\left(Q,K,V\right)=\mathrm{softmax}\left(\frac{QK^{T}}{\sqrt{d_{k}}}\right)V$$

Q, K and V are the matrix of queries, keys and values, respectively. $d_{k}$ is the dimension of the matrices. Furthermore, multi-head attention can be defined as:

(8) $$MultiHead\left(Q,K,V\right)=CONCAT\left({\mathrm{head}}_{1},\ldots ,{\mathrm{head}}_{n}\right)W$$

(9) $${\mathrm{head}}_{i}=\;\mathrm{Attention}\;\left(QW_{i}^{Q},KW_{i}^{K},VW_{i}^{V}\right)$$

After the multi-layer transformer module, we eventually get the final word feature representation $H_{0}^{bert}$.

### 3.5. Co-Attention Layer

**Figure 3:**
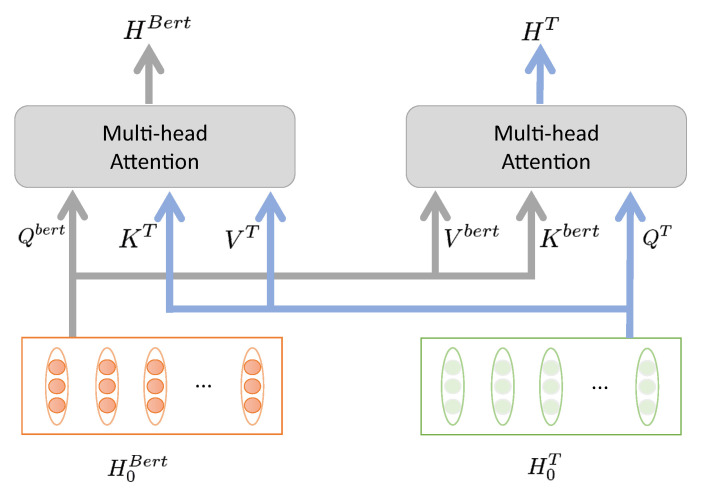
Co-attention layer.

We introduce the co-attention layer as shown in Figure 3. Given the text representation extracted by GNN and Bert, the query, key and value matrices are calculated, just as they are calculated in the standard self-attention mechanism. However, the keys and values of both text features are passed as input to each other’s multi-headed attention block.

According to $H_{0}^{T}$ and $H_{0}^{bert}$, we calculate the query, key and value matrix, respectively. Different from the self-attention mechanism, we take $H_{0}^{T}W_{T}^{Q}$, $H_{0}^{bert}W_{bert}^{K}$ and $H_{0}^{bert}W_{bert}^{V}$ as the input of the formula (7) to obtain $H^{T}$, and take $H_{0}^{bert}W_{bert}^{Q}$, $H_{0}^{T}W_{T}^{K}$ and $H_{0}^{T}W_{T}^{V}$ as the input to obtain $H^{bert}$. Where $W^{Q}$, $W^{K}$, $W^{V}$ are parameter matrices.

Then we get the attention representation of GNN conditioned on Bert output and the attention representation of Bert conditioned on GNN output. Therefore, we obtain the mutually conditional attention convergence feature between the two representations.

### 3.6. Feature Aggregation

We designed three ways to aggregate the extracted features interactively, namely max-pooling, concatenation and addition. For a word $w_{i}$ in the sequence, the features extracted by GNN and Bert are denoted as $h_{i}^{gnn}$ and $h_{i}^{bert}$, respectively. The three aggregation methods are as follows.

**max-pooling**. This function takes the larger value of the two features in each dimension to form the final representation:

(10) $$h_{i}=MAX\left(h_{i}^{gnn},h_{i}^{bert}\right)$$

which chooses the most informative feature in each dimension.

**Concatenation**. It takes the concatenation of the representation directly in the node feature dimension:

(11) $$h_{i}=CONCAT\left(h_{i}^{gnn},h_{i}^{bert}\right)$$

which can keep the output of each module intact.

**Addition**.

(12) $$h_{i}=h_{i}^{gnn}⊕h_{i}^{bert}$$

where ⊕ operation means element-wise addition.

We denote the final representation of the whole document *i* as $H_{i}$.

### 3.7. Final Prediction

After feature aggregation, we employed a fully connected layer for classification. We minimize the cross-entropy loss to train our model.

(13) $$\hat{y}=\mathrm{softmax}\left(WH_{i}+b\right)$$

(14) $$L=-\sum_{i}y_{i}\cdot \log{\hat{y}}_{i}$$

W, b are trainable parameters. ${\hat{y}}_{i}$ and $y_{i}$ are the predicted and true label for the document, respectively.

## 4. Experiments

Here, we evaluated the effect of BEGNN and compared it with baseline models on four publicly available datasets.

### 4.1. Datasets

We adopted four widely used datasets for text classification:

MR [30]. It is a sentiment classification dataset, each review is classified as positive or negative.

SST-2 [31]. It is the Stanford Sentiment Treebank dataset, which includes sentences from movie reviews. Each sample is labeled as negative or positive.

R8 [32]. It is a subset of the Reuters-21578 dataset and had been manually classified into eight categories.

Ohsumed [33]. It is from the MEDLINE database, which is a bibliographic database. Each document had been classified into 23 cardiovascular diseases categories.

The statistics are in Table 2.

For each dataset, we use 10% of the training data for validation to assist in model training. For each piece of data in the dataset, we proceed with it as follows. First, a BertTokenizer is used to segment the document. Second, in the Bert-based feature extraction module, we directly use the segmentation as the input. Third, in the graph neural network-based module, to ensure that the two modules can be aligned, we use the result of Bert word segmentation, and then use the Glove word vector as the words’ initial representation.

### 4.2. Compared Methods

We make a comparison with some state-of-the-art models, including deep models for processing serialized data and models based on GNNs.

Fasttext [34]. A lightweight neural network model. The input is multiple words represented by vectors. In the hidden layer, the average of word vectors is calculated. The last hidden layer’s output is the basis for classification.

Bi-LSTM [35]. It is a kind of RNN. It is specially designed to solve the long-term dependency problem of general RNN. The final hidden state is used for classification.

TextGCN [9]. A GNN based text classification model. The whole corpus is used to construct a large heterogeneous graph. Furthermore, GCN is designed to jointly learn the embedding of words and documents. We build the text graph in the same way as the original paper and use the final representation of the document node as the basis for classification.

TextING [10]. It is another graph based model. Different from TextGCN, it constructs a graph for each text. The final representation of the text is obtained through the output layer and classified.

VGCN-Bert [26]. The word embedding and graph features are fed to the attention layer. Then the attention module’s output is used as the basis for classification.

BEGNN (our proposed method). It is a text classification model combining graph neural networks and Bert, which can extract the semantic and structural information of the text.

Fasttext [34] is a non sequential model while LSTM [35] is a model for sequential data. TextGCN [9] and TextING [10] are graph based models. TextGCN builds a large graph of thesaurus and documents together. The difference is that TextING builds a text graph of words in each document. By comparing these methods, we can analyze which feature is more important to the model.

### 4.3. Hyper-Parameter Settings

Regarding the setting of hyperparameters, based on previous research and experimental experience, we refer to some optimization algorithms based on the Bayesian method [36,37], and use the python open-source toolkit ’advisor’ for parameter optimization.

Regarding the relevant models we compared, we continued the parameter settings of original papers for experiments. For fair comparison, we uniformly use GloVe embedding [15] as the initial word feature vector. For our proposed method BEGNN, we set the learning rate of 0.00005, the l2 regularization weight of 0.01, and the optimized function of Adam. For the text graph, the sliding window size is 3. The number of attention heads is set to 8. Early stopping is applied, the number of epochs is 100. The nonlinearity function is set to ReLU. We use the BertTokenizer to split text. For each dataset, we use three interactive aggregation methods we designed to aggregate the features and report the best results. While training, to ensure the convergence, we firstly pre-trained the GNN network, and then trained the entire model.

### 4.4. Experimental Results

We adopt the classification accuracy and the macro-F1 value as the evaluation metrics. From the experimental results, we can make the following observations. The main results are presented in Table 3.

(1) BEGNN outperforms all the baselines. We use Bert based feature extraction module and GNN based feature extraction module. At the same time, the co-attention module is employed to interactively combine the two features. Suggesting that the combination of GNN based method and pre-trained language method benefits text processing.

(2) The longer the text, the more obvious the improvement of our model to the experimental effect. According to the statistics of the datasets, the text length of R8 and Ohsumed is longer. Especially on the Ohsumed dataset, the average text length is 79. On the datasets where the average text length is less than 20, the performance improvement of our model is relatively lower than the other two datasets with longer text. This shows our model can better process longer texts. Our feature extraction module based on graph neural network passes through message in multiple layers, and can mine the information of multi-hop neighbors. Superior to RNN based model, the self-attention module in Bert can also pay attention to words that are farther away.

(3) RNN based model outperforms Fasttext and TextGCN in two datasets, and shows comparable capability in R8, which shows its advantages in processing sequential data. While in Ohsumed, it does not perform well. The text length of this dataset is long, causing difficulties in processing long-distance context. RNN-based models have no advantage when dealing with longer text data. After long-distance propagation, information will be lost. LSTM adds the memory module to solve the problem of long-distance dependence of traditional RNN architecture, but when the average text length exceeds 70 in the Ohsumed dataset, there are still some problems.

(4) TextGCN and TextING are graph based models. When they are used in text classification tasks, TextING has achieved better results on each dataset. This is because, for the texts, TextGCN constructs a graph of the entire corpus, which is low-density. However, TextING constructs a graph structure for each document separately, which can take into account the different structural information of each text, which will not be so sparse as it in TextGCN.

(5) The performance of VGCN-Bert surpasses other models besides our proposed model. It takes the features extract from graph neural networks and word embedding features as the input of the attention module. However, it builds a graph structure on the entire dataset. Compared with our operation of building a graph structure from a single text, it cannot fully consider the unique structural characteristics of each text. Furthermore, it chooses to concatenate the two representations and send them to the attention module. Different from it, we interact and aggregate the features from GNN module and Bert based module, which can avoid the separation of the two representations and utilize their correlation.

Compared to other related models, first of all, the experimental results demonstrate the superiority of BEGNN. Secondly, our model shows a more obvious advantage in the processing of long texts and can extract features that span longer distances. In addition, our model can take the semantic and structure information of the given documents. The transformer module in Bert uses the attention mechanism to perform parallel calculations, also extracts semantic features. The module based on GNN can extract the structure information of the text well. While the interactive aggregation of these two features can combine the advantages of these two features to the greatest extent. This ensures that BEGNN attains a better effect over the baseline models.

### 4.5. Ablation Study

To analyze the usefulness of each component of BEGNN, we performed the following experiments.

#### 4.5.1. Effectiveness of the Text Graph

**Figure 4:**
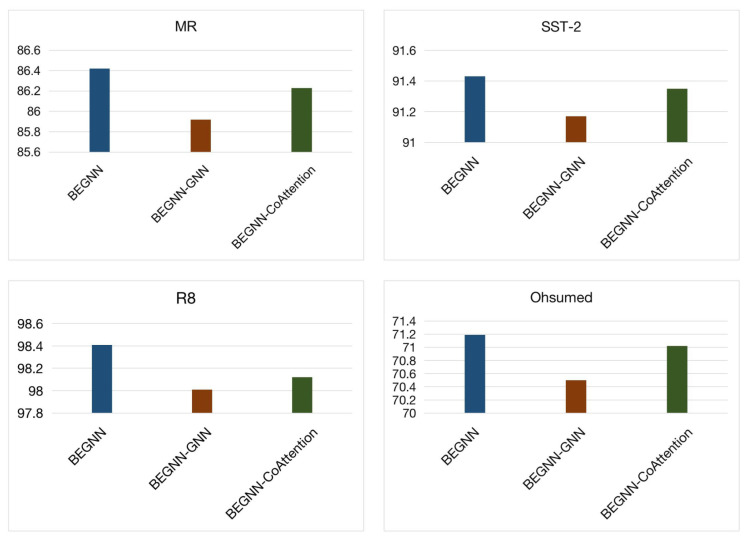
Ablation study of the text graph and the co-attention modules of the model.

In our base model, we build a text graph based on the word co-occurrence relation in the document and aggregate the features obtained from the text graph and the features obtained from Bert. Compared with the original Bert, our model can not only consider the semantic features, but also integrate the structural information. To validate the effectiveness of this module, we designed experiments to compare the effects of our basic model and the model without a graph neural network. We name the model with GNN module removed as BEGNN-GNN. That is, a separate Bert model. Figure 4 illustrates the experimental results. At the same time, we also experimented on the model BEGNN-CoAttention without a feature interaction module.

Compared with using Bert only for training and testing, our original model with graph neural network achieves significant results on four datasets. This confirms the necessity of adding a text graph neural network in our proposed model. Among them, the model with graph structure features has achieved the most significant effect on the Ohsumed dataset. Showing advantages of BEGNN in processing longer text features. Compared with the model without the graph neural network feature extraction module, even without feature interaction, the model containing two granular features still achieves better results than the original Bert model. This also illustrates the importance of adding structural features. Other than semantic features, adding structural features can improve the representation ability of the extracted joint features.

#### 4.5.2. Effectiveness of the Co-Attention Module

On the basis of using Bert to extract semantic features, and adding the structural features extracted by the graph neural network module, we also hope that the two features can interact, rather than being separated from each other. For the features extracted from the Bert model and the graph neural network, we add the co-attention mechanism in order to provide interaction between these two features. We name the model with co-attention module removed as BEGNN-CoAttention.

As shown in Figure 4, removing the co-attention module in the training procedure causes performance degradation on four datasets. In the four datasets, although there is a certain gap in text length, the degree of effect decline is basically the same. When dealing with the interaction of text data, the co-attention mechanism is important in both long and short texts. In the co-attention module, we get the attention representation of GNN conditioned on the features extracted from Bert, and the attention representation of Bert conditioned on the features extracted from GNN. In this way, the two representations interact with each other and improve the performance.

## 5. Conclusions

In this article, we conduct research on text classification algorithms. The application scenarios of text classification are very extensive, and it is important in public opinion analysis and news classification. We propose a Bert-enhanced graph neural network (BEGNN) to improve the representation ability of text. Although it is designed for text classification, its ideas can be applied to other research fields, such as information retrieval. We build a text graph structure for each document and extract the structural features of the text. Furthermore, Bert is used to extract semantic features. In addition, we added an interaction module and aggregated the semantic and structural features of text. Different from other studies, we can take into account the two granular text features in an innovative way, and employ the co-attention module to interact and aggregate them. Experimental results prove the effectiveness of BEGNN.

In future research, we will further study what algorithms and features will have a positive impact on the deep learning model when using Bert and graph neural network for feature extraction. At the same time, we will study how to use this analysis result to further optimize the model, increase the interpretability of the model and produce more fine-grained and reasonable interpretation. We will also consider further research on the lightweight optimization to reduce the cost of calculation and reasoning while ensuring the effect of the model.

## Figures and Tables

**Table 1:** Comparison of some related work.

Related Researches	Method
[7]	Self-encoding language model, constructed using the encoder module of transformer.
[20]	Explore how to fine-tune Bert to improve the effect of text classification, including hierarchical learning rate adjustment, multi-task pre-training and other methods.
[21]	Prove the superiority of bert over traditional machine learning algorithms.
[9]	Build a heterogeneous graph for the entire dataset, and use the representation of the document node as the classification basis.
[10]	Build a separate graph for each text, use graph neural network and design the output layer to get the representation and classification of the text.
[26]	Build a graph for the entire dataset, word embedding and graph feature are feeded into attention layer.
[24]	Build a graph for the entire dataset, integrate the graph convolution features of multi-hop neighbors.
[28]	The output of GNN and Bert are concatenated for the recommendation task.
[18]	Dynamically Gated Convolutional Neural Network.
[25]	Research on effect of different embedding technologies when they are used together.

**Table 2:** Statistics of datasets.

Dataset	Documents	Training	Test	Class	Average Length
MR	10,662	7108	3554	2	18
SST-2	9613	7792	1821	2	19
R8	7674	5485	2189	8	41
Ohsumed	7400	3357	4043	23	79

**Table 3:** This is a table caption. Tables should be placed in the main text near to the first time they are cited.

Methods	MR	SST-2	R8	Ohsumed
Fasttext	75.04	80.25	96.11	57.70
Bi-LSTM	76.27	81.23	96.30	50.27
TextGCN	75.56	80.25	96.89	67.44
TextING	78.93	83.69	97.92	70.41
VGCN-BERT	86.21	91.02	97.98	70.53
BEGNN	86.42	91.43	98.41	71.19

## Data Availability

The source code and the datasets used in the experiments is available at https://github.com/pingpingand/BEGNN, accessed on 24 July 2021.
